# Application of Recurrent Neural Network Algorithm in Intelligent Detection of Clinical Ultrasound Images of Human Lungs

**DOI:** 10.1155/2022/9602740

**Published:** 2022-06-24

**Authors:** Yanjie Jia

**Affiliations:** Affiliated Hospital of Chifeng College, Chifeng 024000, Inner Mongolia, China

## Abstract

Lung ultrasound has great application value in the differential diagnosis of pulmonary exudative lesions. It has good sensitivity and specificity for the diagnosis of various pulmonary diseases in neonates and children. It is believed that it can replace chest CT examination. It is routinely used for the diagnosis of pulmonary diseases in emergency critical care medicine. However, the interpretation of the impact of ultrasound on the human lungs relies heavily on experienced physicians, which greatly restricts the interpretation efficiency of the impact of ultrasound. In order to improve the efficiency of monitoring and interpretation of the impact of ultrasound, this paper proposes an intelligent detection algorithm for human lung clinical ultrasound images based on recurrent neural network. Transfer learning is used to replace the fully connected layer of the VGG16 model and improve the loss function, so that the same the Euclidean distance between category images can be reduced, and the Euclidean distance between different categories of images can be increased, enhancing the resolution of the entire model, thereby achieving better image feature extraction results. The experimental results show that the algorithm proposed in this paper can surpass the doctor's level in the identification of various lung diseases.

## 1. Introduction

The lungs are the main air-bearing organs of the body. Due to the physical characteristics of ultrasound itself, when the propagation path encounters gas, total reflection will occur, resulting in ultrasound not being able to capture the image of the normal lung tissue. In recent years, with the continuous development of ultrasound technology, ultrasound diagnosis of lung diseases has become an important means to check and monitor the treatment effect in the world, realizing the visualization of lung pathophysiology, known as the visual “stethoscope” [1].

With Lichtenstein's discovery of the value of pulmonary ultrasound, the establishment of perfect operating and scoring rules, and the formation of the imaging theory and procedure of LUS, LUS has become a simple and reliable means of diagnosing pulmonary diseases in recent years. The International Consortium of Lung Ultrasound has issued an international consensus on the diagnosis of lung diseases by ultrasound [2]. It is believed that lung ultrasound can not only accurately diagnose many lung diseases but also has higher accuracy and sensitivity than chest X-ray in the diagnosis of pneumothorax, pulmonary solids, and pleural effusion.

The determination of the type of lung disease relies mainly on the physician's subjective diagnosis of the patient's lung images. The accuracy of the diagnosis of lung diseases depends on the physician's personal condition and is highly subjective [3]. Moreover, the orientation of certain lung lesions varies from person to person, and the lung structure varies greatly from person to person. Therefore, it is also easy to create factors that may produce errors in this regard, thus failing to meet the requirements of precision medicine [4]. On the other hand, not all regions have good medical resources and medical conditions, and the medical conditions enjoyed by patients in different regions may be very different [5]. Computer-aided medical image recognition and diagnosis is able to overcome these difficulties and solve the current problems. Computer-aided medical image recognition and diagnosis aims to improve the accuracy of diagnosis by quickly and accurately detecting lesions.

This paper studies the theory of optimization on the one hand, will be conducive to the algorithm, On the other hand, it is beneficial to promote the efficiency of pulmonary ultrasound detection and has certain theoretical and application significance.

## 2. State of the Art

When it comes to image recognition, it is inseparable from the convolutional neural network of deep learning. Convolution neural network is a kind of feedforward neural network with convolution computation and depth structure. It is one of the representatives of the depth learning algorithm. It goes beyond the traditional learning tools and methods. Convolutional neural network, which is called neural network, plays a central role in the field of medical image recognition and image recognition. We have to mention the original convolutional neural network LeNet.

The proposal of LeNet marked the birth of CNN, and the 7-layer LeNet contains the basic modules of deep learning. The simple network is capable of automatic recognition of handwritten recognition numbers and automatic verification of CAPTCHA. AlexNet, designed by Hinton and his student Alex Krizhevsky, was the winner of the ILSVRC competition. Several technical breakthroughs and applications of new technologies were achieved in the AlexNet network. It is these techniques that take convolutional neural networks to new heights [6].

The innovations used in AlexNet include the following: (1) The use of the Relu activation function instead of the original Sigmoid activation function. When the number of layers of the neural network increases to a certain depth, if the Sigmoid activation function is used, the gradient will disappear and the whole network will converge too slowly, or there will be a gradient dispersion problem, using the Relu function is a good solution to this problem [7]. (2) The Dropout function is used during training, through which the parameters of some neurons can be randomly ignored, i.e., the activity of some neurons is suppressed, and this measure can also avoid overfitting of the model [8]. (3) Applying overlapping pooling layers to the convolutional neural network can not only significantly improve the computational speed compared with the previous average pooling layers but also—to a certain extent—reduce the overfitting of the model [9]. The overfitting of the model can be reduced to a certain extent. (4) The dataset expansion technique is used. (5) CUDA acceleration is used to reduce the computation time so that the number of layers of the neural network can be deepened to improve the overall model recognition accuracy. (6) The concept of LRU was introduced for the first time, adding LRU layer to CNN, the role of which is to filter the neurons in the network model layer, making the value of neurons with larger responses larger and the value of neurons with smaller responses smaller, which can well suggest the robustness and generalization ability of the model.

Since then, convolutional neural networks have developed rapidly and VGG series networks have emerged. The network structure of VGG16 is shown in Table 1. The advantage of VGG series is that the structure of the whole neural network is very simple, which can improve the calculation speed. Secondly, VGG series through the test of its own data, verified by deepening the depth of the neural network, improves the network identification performance, and can improve the accuracy of identification. Table 1 describes from top to bottom the number of neurons in VGG16 neural network layer, the arrangement of convolutional layer and pooling layer, and the size of the input and output image feature matrix of each neural network layer.

The core neural network representative of GoogLeNet is the Inception network, which is designed as a net-within-a-net structure, giving the model the ability to choose itself, with multiple choices for each layer, and the model automatically adjusts to choose the layer with higher recognition accuracy for training recognition. By adding 1 × 1 convolutional kernel layer, the whole Inception network can be designed to be deeper and the width can be expanded and adjusted, and the overall effect can reach 2–3 times of the original performance. The error rate of recognizing the top5 classified images was only 6.67%.

The biggest innovation highlight of Inception Net is the design of Module module. Module brings improvement not only in the overall model performance but also reduces the number of image data parameters. It provides the ability of the model to cross channels, improves the information transfer and expression ability of the network, and provides the model with more choices of linear transformation types. On the other hand, it is a regularization method that is first proposed and applied in the model for the whole network, which speeds up the convergence and improves the overall classification and recognition accuracy of the model [10]. The data are normalized in the implementation process to limit the range to *N* (0, 1). The ResNets (residual networks) that emerged afterwards extend the depth of the network to several hundred layers by a special computational approach. This method is based on the idea of Highway Network, which opens up a “high speed channel” in the process of network propagation and is computed in an optimal fitting way [11].

Based on previous algorithm studies, the advantages of convolutional neural network in image recognition can be seen. Compared with other neural networks, the network has better fitting degree and higher accuracy rate of image recognition, so it can be better applied in intelligent detection of human lung clinical ultrasound images.

**Figure 1:**
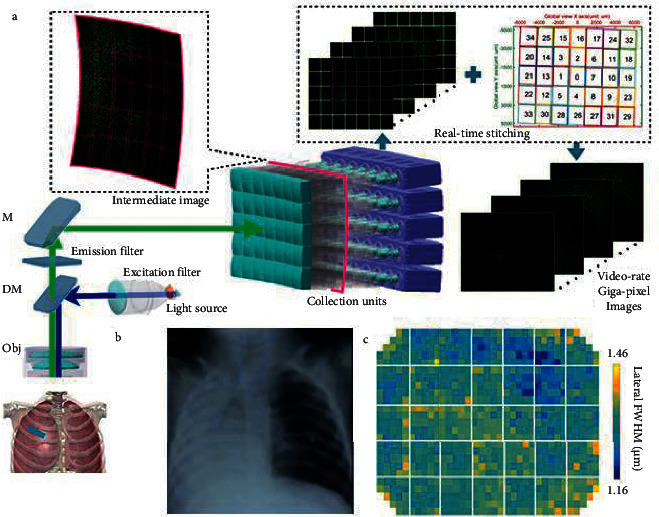
Algorithm structure diagram.

By combing the previous development context, we can see that the optimization of the past is a dark mass of optimization in the input layer. In order to take advantage of deep learning to interpret ultrasound images of lungs, we proposed a neural network algorithm based on VGG16, whose basic structure is shown in Figure 1. We replace the output layer of THE VGG16 neural network with the ultrasound image of the lung as the initial input, so as to improve the accuracy of the neural network results, and use the transfer learning method to make the VGG16 neural network more suitable for lung ultrasound interpretation.

## 3. Methodology

### 3.1. Data Set

It is difficult to obtain medical images of lungs, and there are no datasets of lung medical images of the order of 10 million or millions at present. This is also a problem at present, and the solution is to expand the image dataset.

**Figure 2:**
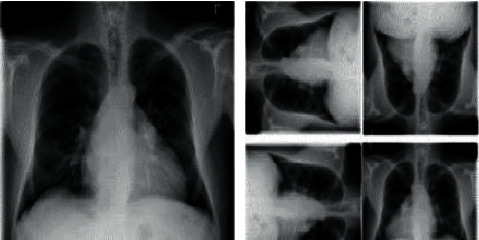
Lung image dataset expansion.

In order to ensure the integrity of the image data, ensure the scientific nature and authenticity of the experiment. The external library PIL in Python was used to realize the extension of lung medical image, and the image recognition and classification effects such as image rotation, mirror image, brightness adjustment, chroma adjustment, and random cropping were compared. Finally, image rotation and mirror were selected as the extension method of lung medical image dataset [12]. Because the whole model structure is divided into five branches, it is selected to use the original image to generate three image rotation directions, and then generate a mirror of a total of five pictures, that is to say, one original image will be extended to five, input different branches as the input images, the dataset expansion result is shown in Figure 2.

The purpose of dataset enhancement is mainly to perform some transformations on the input image data and train a model with better robustness, so that it can cope with various transformations and be more applicable to real scenes. For example, when taking pictures in life, the angle, brightness, stretching, etc., of the pictures obtained may be different due to different shooting locations or different cameramen, whether or not the different images can be recognized as the same structure. Again performing dataset enhancement is to train the model to be able to achieve excellent recognition accuracy with so many transformations [13]. AlexNet does a good job of dataset augmentation and eventually achieves good results.

There are many ways to enhance image datasets: one common method is to apply a bevel attribute to the image, and another is to add a shrink and expand factor.

The interface to the dataset enhancement algorithm is integrated in the Python external library Keras, which can be easily invoked by setting the corresponding parameters [14].

### 3.2. Recurrent Neural Network

Recursive neural network is an artificial neural network with tree-like hierarchical structure and the network nodes recurse the input information according to their connection order, which is one of the deep learning algorithms. Recursive neural networks can be trained using supervised and unsupervised learning theories. In supervised learning, recursive neural network uses back propagation algorithm to update weight parameters, and the calculation process can be similar to the back propagation algorithm over time of recurrent neural network. Unsupervised learning recursive neural networks are used for representational learning of structural information and the most common form of organization is recursive self-encoder.

For a recurrent neural network, it is a network where the current output is related to the previous output as well. This means that the network remembers the last output result and then uses it in the calculation of this output, which makes the nodes between the intermediate layers transform from a connectionless one to a connected one, and the input given by the intermediate layers has both the output of the input layer and the output of the intermediate layer at the last moment.

**Figure 3:**
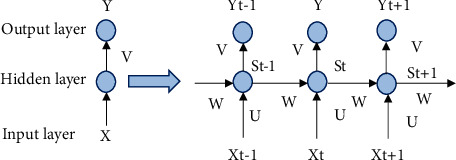
Structural diagram of recurrent neural network expansion.

**Figure 4:**
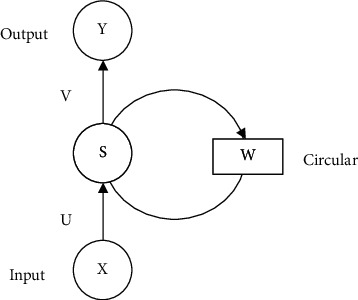
Recursive neural network structure.

Where *X* represents the input vector, *U* represents the weight matrix from the input to the intermediate layer, similarly *W* represents the weight matrix from the state to the intermediate layer, *S* is the state value of the node in the intermediate layer, *V* represents the weight matrix from the loop layer to the output layer, and *Y* represents the output. The value of state *S* is not only output to the output layer but also calculated by multiplying *W* and then used as input for the next calculation [15].  Figure 3 shows the expansion diagram of the recurrent neural network expanded along time in Figure 4.

Where the network input at time *t* is *X*_*t*_, St denotes the value of the intermediate layer, and the output is *Y*_*t*_. It is clear that the value of *S*_*t*_ depends not only on *X*_*t*_ but also on *S*_*t*_ − 1 as well. They can be expressed in equations as shown in (1) and (2), where *g* and *f* denote the activation functions.

(1) $$\begin{array}{c} Y_{t}=g\left(V_{t}S_{t}\right), \end{array}$$

(2) $$\begin{array}{c} S_{t}=f\left(UX_{i}+W_{t}S_{t-1}\right). \end{array}$$

Compared with other neural networks, the structure of recursive neural network can well introduce the concept of time into the neural network, which can make the input data at the previous moment directly affect the data calculation at that moment. St state can remember the information at the previous moment. Thus, it is beneficial for real-time image recognition. Therefore, this paper chose recursive neural network for pulmonary clinical image ultrasound detection. In practice, however, with the increase of depth of the model, the recursive neural network cannot handle well data dependency for a long time, so you can understand, the so-called back-propagation gradient disappeared and gradient caused by explosion problem, so this article think should optimize the recursive neural network so as to solve the data dependency problem for a long time. To solve these problems, an improved recursive neural network, LSTM, is proposed.

LSTM adds three gate selection units in the recursive neural network, which solves the problems of gradient disappearance and gradient explosion of ordinary RNN (existing neural network), and forms an independent transmission mechanism for memory data and result data, so that information can be transmitted across regions. Overcomes the problem that standard RNN does not handle data dependency well over time. Deals with long time dependencies on data. Therefore, LSTM is one of the most popular recursive neural networks and has been widely used in several fields of artificial intelligence [16].

**Figure 5:**
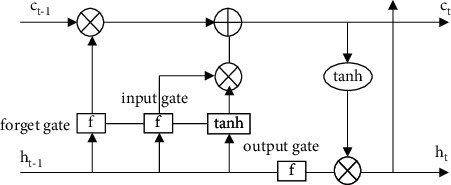
Structure of LSTM network.

Figure 5 shows the structure of LSTM network, which has four network layers that make it different from the normal RNN network, LSTM stores the information selectively, while the normal RNN stores the information whether it is useful or not. The gates are sigmoid and tanh functions, and the process is implemented by multiplying the neural layers of the sigmoid or tanh functions with each point to make it possible [17]. The gates in this network are the forgetting gate and the input and output gates, respectively.

Where ⊗ denotes the dot product, *f* denotes the activation function, *C*_*t*_ − 1 denotes the cell state at the previous time step, *h*_*t*_ − 1 denotes the output value at the previous time step, ct is the cell state at this moment, and ht denotes the output value at this moment.

(1) forget gate is a judgment of what information is discarded by the cell state, and this judgment is done by the sigmoid function, expressed as shown in the following equation:
  (3) $$\begin{array}{c} f_{t}=\sigma \left(W_{f}\left[h_{t-1},x_{t}\right]+b_{f}\right), \end{array}$$
where *ht* − 1 denotes the last—moment output value, *x*_*t*_ denotes the input value at this moment, Wf denotes the weight matrix of this gate, *b*_*t*_ denotes the bias of this gate, and *σ* denotes the activation function.
(2) The input gate (input gatc) determines what information is stored in the cell state. First, the input gate is called a sigmoid layer, and second, a vector is created by calling the tanh layer as a new marquee value that can be added to the intermediate layer states [18]. Finally, the two parts are merged to create an update to the hidden layer state, which can replace the old memory information forgotten, then represented as shown in equations (4) and (5):
  (4) $$\begin{array}{c} i_{t}=\sigma \left(W_{i}\left[h_{t-1},x_{t}\right]+b_{i}\right), \end{array}$$
  (5) $$\begin{array}{c} {\tilde{c}}_{t}=\tanh\left(W_{c}\left[h_{t-1},x_{t}\right]+b_{c}\right), \end{array}$$
In this case, if we know the memories to be forgotten and those to be added, we can update the old cell state *c*_*t*_ − 1 to the new cell state ct. After that, the old m-server state will be multiplied by FT, which will determine the information to be forgotten, and then multiplied by the sum of the input gate of the current memory *c_t_* [19]. After this series of operations, the new memory information will be derived, which is expressed as shown in the following equation (6):
  (6) $$\begin{array}{c} c_{t}=f_{t}*c_{t-1}+i_{t}*{\tilde{c}}_{t}. \end{array}$$
(3) The output gate is the information that needs to be decided for the output. Although the output is based on the cell state, it will be a post-selection value. This output gate needs to go through three steps to get the output information [20]. First, the output gate needs to go through a sigmoid layer, second, a partial selective output is performed for different parts of the cell state, and finally, the obtained cell state is fed into the tahn activation function so that it is multiplied with the sigmoid activation function to obtain a value that needs to be output, which is represented as shown in (7) and (8):
  (7) $$\begin{array}{c} o_{t}=\sigma \left(W_{o}\left[h_{t-1},x_{t}\right]+b_{o}\right), \end{array}$$
  (8) $$\begin{array}{c} h_{t}=o_{t}*\;\tanh\left(c_{t}\right), \end{array}$$
where *b*_*o*_ denotes the output bias, *W*_*o*_ denotes the number of weights of the output, *h*_*t*_ denotes the output value of the cell state, and ot denotes the value of the output gate.

## 4. Result Analysis and Discussión

### 4.1. Principle of VGG16 Extraction of Image Features

Before introducing VGG16, it is necessary to introduce the general convolutional neural network computational rules and the important parts of it. The more important components of the convolutional neural network are the convolutional layer, the pooling layer, and the activation function, and the computational method is the BP back propagation algorithm.

**Figure 6:**
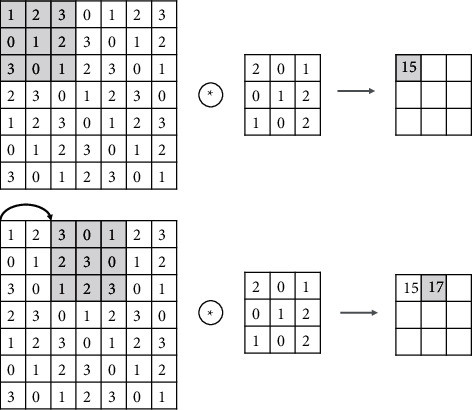
Convolution kernel to extract image features.

The convolutional layer plays a crucial role in the whole neural network layer, which is equivalent to a filter, and consists of convolutional kernels, the number and size of which are set by the designer of the network, generally 3 × 3, 5 × 5, 7 × 7, 11 × 11. According to the set size of the convolution kernel, a weighted average calculation is performed on the corresponding size area of the input image. Depending on the step size, the size of the convolution kernel, and the setting of the convolution algorithm, the convolution process will extract different numbers of image features, and the final output matrix is the output of the convolution kernel on the image. The final convolution value is obtained by multiplying the convolution kernel parameters with the image pixel values in the matrix, and then the length of each convolution kernel move is determined by the convolution kernel step setting. After the convolution, kernel shift calculation will reduce the size of the whole image feature matrix, as seen in Figure 6.

The pooling layer is used to reduce the number of features extracted by the convolutional layer, i.e., to reduce the size of the feature matrix, thus reducing the system resources and time consumed during the model computation, and is also useful in preventing overfitting. This process can also be called “downsampling.” There are two main pooling layers: the maximum pooling layer, which extracts the largest value in the pooling matrix as the output, and the average pooling layer, which extracts the average value of the parameters in the pooling matrix as the output.

The activation function is set in each layer of the network, and each layer has input and output data. The input data are the parameter value transferred from the upper layer after the calculation, and the output value obtained after the calculation of the specific function is used as the input value for the lower layer of the network. The specific function in the middle refers to the activation function. There are many different types of activation functions. In order to make the values calculated by the activation function more nonlinear, the following activation functions are commonly used.

After the network reaches the last layer, the difference between the predicted result and the real result is calculated according to the corresponding loss function, according to which the BP back propagation algorithm goes forward to adjust the parameter values of the whole network model, so that the model can obtain better recognition and classification results.

The structure of the VGG16 model contains 13 convolutional layers, 3 fully connected layers, and 5 pooling layers. After training, the complete model is able to extract features from images.

### 4.2. Single-Branch Model Structure

In this paper, gradient advancing tree in traditional machine learning model is selected as the classification model of lung image recognition. The gradient advancing tree model is suitable for the classification and recognition scenes with small datasets. However, the traditional machine learning model requires manual extraction of image features on the one hand, and the upper limit of classification and recognition accuracy on the other hand is not very high. In order to solve these problems, the method commonly adopted abroad is to modify the classical model a lot, until it is applicable to the size of the corresponding dataset; using this method will cause a great loss of the ability of the VGG16 model to extract image features. Therefore, in the research method of this paper, we first use migration to adjust the parameters of the VGG16 model. Migration research is a machine learning method, which starts from a model in the development task. The model used in the development process is task B. The migration research method in this paper can improve the efficiency of model development. In this paper, the loss function of VGG16 is improved, the clustering effect is increased, and the ability of the whole model to extract image features is improved. Then, the function of the first half of the model is to extract the features of lung medical images, and then input the extracted features into the gradient enhanced tree model for recognition and classification.

In this paper, we modify the fully connected layer of the original VGG16 model by using the idea of migration learning, removing the fully connected layer, and replacing the fully connected layer with a fully connected network of our own design, and modifying the corresponding activation function and dropout parameters. The second and third layers are set to 256 neurons, respectively, reducing the number of neurons in each layer so that the training of the whole model can match the number of images in the training set and avoid the effect of overfitting the model. The loss function of the original classical model is innovatively improved to include clustering effect, so that the feature distance between images of the same category is reduced, while the feature distance between images of different categories is increased, thus improving the recognition and classification ability of the model. The image features extracted by the neural network are input to the gradient boost tree model for recognition and classification.

The first step of training the single-branch model is to obtain the lung medical image dataset and image preprocessing, after which the fully connected layer of VGG16 is replaced and the parameters are fine-tuned using migration learning, with the conflict of needing to extract image features manually. On the other hand, the single-branch model has certain advantages in terms of computational speed and computational efficiency because only one branch of training computation is required. However, the disadvantage of single-branch model is that the accuracy of recognition and classification may fluctuate greatly due to different scenes or different image datasets, which causes the stability and robustness of the model to be low and thus cannot be applied to the training recognition and classification of different scenes and different datasets; so we consider designing multiple-branch model structure and combining the recognition results of each branch through corresponding algorithms to finally complete the image. The model structure of multiple branches is considered, and the recognition results of each branch are combined by the corresponding algorithm to complete the classification of images.

### 4.3. Migration Learning of VGG16

The purpose of migration learning is to preserve the ability of VGG16 to extract features from images. Because of the small number of image datasets, the training cannot be done from scratch based on the original model, but rather fine-tuning the parameters for certain layers.

VGG16 consists of 13 convolutional layers, 5 pooling layers, and 3 fully connected layers. The changes in image size and number of channels per layer in the original and modified model structures are shown in Figure 6. The number of neurons in each of the three replacement layers is set to 4096, 256, and 256, respectively. The activation function uses the Relu function, which is called to splice the first 15 layers of the classical model with the fully connected layers designed by itself, and the parameters of the training layers can be adjusted so that the whole model is involved in the training, but only the parameters of the fully connected layers designed by itself are adjusted.

The first 15 layers of the VGG16 model have not been changed, but only the last three fully connected layers have been changed. The number of neurons in the fully connected layer is reduced and the corresponding activation function and dropout parameters are changed, the regularization coefficients are changed, and the training is performed using medical images of lungs. The training process only adjusts the three-layer model designed by ourselves, which can be set by the trainable parameter in the layer external library to avoid the participation of the first half of the VGG16 model in the training.

### 4.4. Model Accuracy and Robustness Verification and Analysis

The main objective of this research method is to solve the following problems: firstly, the VGG16 model is improved and fine-tuned by transfer learning to resolve the contradiction between the complex VGG16 model and insufficient lung images; secondly, the loss function in the VGG16 model is improved to include the judgment of inter-class similarity of lung images to make the model have the effect of clustering; after that, the VGG16 extracted image. Finally, the above model is used as a whole to combine multiple branches into a strong classifier by weighted voting or majority voting method to enhance the accuracy, precision, and recall of model recognition and improve the recognition ability and robustness of the model.

**Figure 7:**
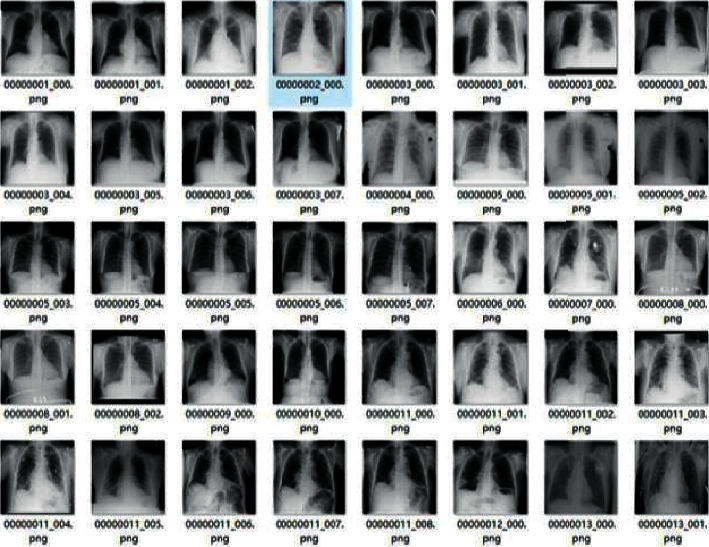
Partial dataset images.

The data used in this study are from the publicly available ChestX-ray14 dataset, which is provided by https://www.kaggle.com/nih-chest-xrays/data. Images of five diseases (Atelectasis, Emphysema, Infiltration, Nodule, and Pneumonia) from the ChestX-ray14 dataset were selected. Some of the images in the dataset are shown in Figure 6. After that, the original images were rotated by 90°, 180°, and 270° and mirrored to obtain 4 patch images, thus expanding each 1 original image to 5. The number of images is then expanded by using data enhancement methods including image scaling, beveling, and panning. All images are converted to a uniform format and size and randomly divided into a training set and a test set (ratio 8 : 2). The recognition accuracy of the training set and the comparison results of the loss during training are shown in Figure 7.

From the accuracy graph, we can see that CheXNet starts to improve from 78%, while the model designed in this paper starts to improve from 65%, indicating that the dataset trained by CheXNet before is very similar to the lung images, while the initial dataset of the VGG16 model used in this paper is mostly images from daily life, which is different from the medical images.So the recognition accuracy of the model at the beginning will be improved. However, as the number of training iterations increases and the recognition accuracy of the model remains stable, the model designed in this paper clearly outperforms the recognition accuracy of CheXNet, and this conclusion can also be confirmed in the comparison of the loss results during training.

In the comparison graph of model recognition accuracy, my_acc represents the recognition accuracy of the model designed in this paper, and standard_acc represents the recognition accuracy of CheXNet; in the comparison graph of loss during training, my_loss represents the change of loss during the iteration of the model designed in this paper, and standard_loss represents the change of loss during the iteration of the CheXNet model. my_loss represents the change of loss in the iterative process of the design model, and standard_loss represents the change of loss in the iterative process of the CheXNet model. In the comparison graph of recognition accuracy, when the recognition accuracy curve is close to smooth, the recognition accuracy of this design model is significantly better than that of CheXNet model; in the comparison graph of loss calculated during iteration, when the loss curve is close to smooth, the loss value calculated during iteration of this design model is lower than that calculated during iteration of CheXNet. The loss value calculated in the iterative process of the model designed in this paper is lower than that calculated in the iterative process of CheXNet when the loss curve is close to smooth. The loss here refers to the loss result value calculated by the loss function in each iteration of the model training process. By observing the loss value change graph, we can also analyze the advantages and disadvantages of different models in terms of recognition accuracy.

After the trained model is obtained, the recognition accuracy of the model is verified using the test set images obtained during image preprocessing, and the final accuracy verification conclusion is obtained by comparing the recognition accuracy of different models for the test set lung images, i.e., the model-assisted lung image disease classification accuracy.

**Figure 8:**
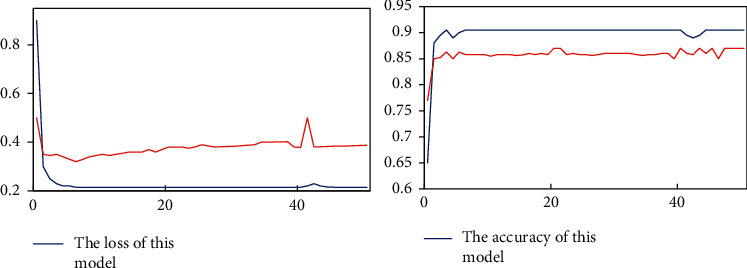
Comparison of training results.

Using the trained model for classification recognition on the test set, the test results are shown in Figure 8. In the comparison graph of recognition accuracy results, the CheXNet model and the model designed in this paper are tested and compared, and it is obvious that the model recognition accuracy is better or worse.

The blue curve in the figure shows the accuracy of the model designed in this paper in recognizing the test set data, and the red curve shows the accuracy of the CheXNet model in recognizing the test set images. It is obvious from the images that the model designed in this paper has better prediction effect than the current advanced CheXNet model.

In this paper, the different lung disease recognition accuracies were extracted separately and compared with the previous models. In this paper, the data from the original literature was selected and a row was added to include the data of the accuracy of the single lung disease recognized by the model designed in this paper for comparison with the previous models, as shown in Table 2. From the table, we can see the recognition accuracy of different models for each of the five lung images to be classified individually, which can also verify the advantages of the model designed in this paper in terms of recognition accuracy.

The robustness of the model designed in this paper was verified by comparing the accuracy and recall of the model designed in this paper with the CheXNet model and the F-values calculated by radiologists for image classification of five lung diseases, and the comparison results are shown in Table 3. It can be seen that the model in this paper has higher recognition accuracy.

## 5. Conclusion

In recent years, with the continuous development of ultrasound technology, ultrasound diagnosis of lung diseases has become an important means to check and monitor the treatment effect in the world, realizing the visualization of lung pathophysiology, known as the visual “stethoscope.” In order to improve the recognition efficiency of lung ultrasound images, this paper proposed a lung image classification recognition method combining convolutional neural network VGG16 and gradient enhanced tree model. In order to improve the efficiency of model optimization, transfer learning is used to replace, train, and fine-tune the full connection layer of the VGG16 model. The original loss function is improved, and a new loss function is proposed, which reduces the Euclidean distance between the same category images and increases the Euclidean distance between different categories images, so as to enhance the recognition ability of the whole model and achieve better image feature extraction. Finally, multiple branches are designed and the classification results of these branches are combined by majority voting algorithm or weighted voting algorithm to obtain the detection results of lung images. It can be seen from the results that compared with the algorithm before optimization, the algorithm model designed in this paper has higher precision and accuracy.

## Figures and Tables

**Table 1:** VGG16 structure.

ConvNet configuration					
A	A-LRN	B	C	D	E
11 weight layers	11 weight layers	13 weight layers	16 weight layers	16 weight layers	19 weight layers

Input (224 × 224 RGB image)					
conv3-64	conv3-64	conv3-64	conv3-64	conv3-64	conv3-64
	LRN	conv3-64	conv3-64	conv3-64	conv3-64

Maxpool					
conv3-128	conv3-128	conv3-128	conv3-128	conv3-128	conv3-128
		conv3-128	conv3-128	conv3-128	conv3-128

Maxpool					
conv3-256	conv3-256	conv3-256	conv3-256	conv3-256	conv3-256
conv3-256	conv3-256	conv3-256	conv3-256	conv3-256	conv3-256
			conv1-256	conv3-256	conv3-256
					conv3-256

Maxpool					
conv3-512	conv3-512	conv3-512	conv3-512	conv3-512	conv3-512
conv3-512	conv3-512	conv3-512	conv3-512	conv3-512	conv3-512
			conv1-512	conv3-512	conv3-512
					conv3-512

Maxpool					
conv3-512	conv3-512	conv3-512	conv3-512	conv3-512	conv3-512
conv3-512	conv3-512	conv3-512	conv3-512	conv3-512	conv3-512
			conv1-512	conv3-512	conv3-512
					conv3-512

Maxpool					

FC-4096					
FC-4096					
FC-1000					
Soft-max					

**Table 2:** Comparison of accuracy rates of different models for lung diseases.

Disease type	Models			
	Wang et al., (2017)	Yao et al., (2017)	CheXNet models	Text model
Pulmonary atelectasis	0.716	0.772	0.8094	0.8243
Pulmonary emphysema	0.815	0.829	0.9371	0.9537
Pulmonary infiltration	0.609	0.695	0.7345	0.8211
Tuberculosis	0.671	0.717	0.7802	0.7981
Pneumonia	0.633	0.713	0.7680	0.7813

**Table 3:** Model robustness comparison.

Models	Standard		
	Accuracy rate	Recall rate	F-value
Physician average	0.330	0.442	0.378
CheXNet average	0.387	0.481	0.428
The model in this paper averages	0.415	0.496	0.452

## Data Availability

The labeled dataset used to support the findings of this study is available from the corresponding author upon request.
